# Beyond text: Using network centralities and AI models to detect suicide risk on Reddit

**DOI:** 10.1016/j.ijchp.2026.100705

**Published:** 2026-06-25

**Authors:** Golnaz Nikmehr, Aritz Bilbao-Jayo, Aitor Almeida

**Affiliations:** Deustotech, University of Deusto, Bilbao, Spain

**Keywords:** Social networks, Suicidal ideation detection, Deep learning, Machine learning, Centrality, Social media, Graph neural networks

## Abstract

Suicide prevention research increasingly leverages social media data, where individuals often share personal experiences and emotions. While most studies rely on textual analysis to detect suicidal ideation, the structural characteristics of online social networks remain underexplored. In this exploratory pilot-scale study, we investigate whether network centrality metrics derived from Reddit interaction graphs can provide complementary signals for suicide risk detection beyond text-centric approaches. We construct directed user interaction graphs and extract 14 centrality metrics, including degree, betweenness, eigenvector, authority, and Katz centralities, to evaluate their predictive power. Using traditional machine learning classifiers, we assess the performance of these graph-based features in distinguishing suicidal from non-suicidal users. To further explore the interplay between network structure and textual content, we integrate centrality features with Sentence-BERT (SBERT) embeddings within a Graph Neural Network (GNN) framework. Within the constraints of a small-scale dataset, the best-performing GNN with SBERT achieved 67% accuracy and a 67% F1-score, representing a marginal improvement over the Random Forest baseline (64% accuracy; F1 = 66%). Results show that network centralities alone provide meaningful signals of suicidal risk, revealing patterns of isolation, influence, and social connectedness that characterize vulnerable users. Although these findings provide preliminary evidence for the complementary value of structural network analysis, they should be interpreted cautiously due to dataset scale and keyword-based sampling constraints.

## Highlights

• Presents an exploratory pilot-scale analysis of Reddit network centralities for suicide risk detection.
• Shows structural features may provide preliminary predictive value independent of textual content.
• Compares ML and GNN models using graph and text embeddings.
• Finds centralities reveal patterns of isolation and influence.
• Highlights both the promise and methodological limitations of network-based mental health monitoring.

## Introduction

Suicide is a major global public health issue, claiming over 720,000 lives annually and ranking as the third leading cause of death among individuals aged 15–29 ((World Health Organization, 2024)). Both the World Health Organization and the (American Association of Suicidology, 2023) define suicidal ideation as “thoughts, considerations, or plans related to suicide,” encompassing both passive ideation (thoughts of death without plans) and active ideation (concrete plans and intentions). Suicidal ideation arises from a complex interplay of mental health conditions, traumatic experiences, and environmental stressors, highlighting its multifaceted nature.

The rise of social media platforms such as Reddit has created new opportunities to study expressions of suicidal thoughts. Individuals often share personal reflections, seek advice, or discuss life after death, with platform anonymity enabling more candid communication. Most existing research focuses on textual analysis of posts, using traditional machine learning or deep learning methods to detect suicidal ideation (e.g., (Burnap et al., 2017); (Sawhney et al., 2021)). While effective, these approaches can miss users who express distress implicitly or through their social interactions rather than textual content.

Recent advances in artificial intelligence have created new opportunities for scalable health monitoring and decision support while also highlighting the need for careful evaluation, regulation, and responsible deployment in healthcare settings (Angus et al., 2025). This perspective is particularly relevant for suicide-risk detection, where algorithmic tools may support the early identification of individuals at risk but should not be viewed as substitutes for clinical assessment and human judgment.

In contrast, social network structures provide complementary insights. Graph-based features, such as user connectivity, interaction patterns, and centrality measures, can reveal hidden behavioral signals associated with suicidal risk. Prior studies have highlighted the importance of social connectedness for mental health, with isolation and reduced support linked to increased vulnerability ((Wickramaratne et al., 2022)). However, the potential of network centralities for predicting suicidal behavior remains underexplored.

This study addresses this gap by investigating the predictive power of network centrality measures derived from Reddit user interaction graphs. We analyze 14 centralities to identify the most informative metrics and assess their utility in detecting at-risk users. Furthermore, we explore the integration of structural and textual information using Graph Neural Networks (GNNs) with Sentence-BERT (SBERT) embeddings to enhance suicide risk detection.

The primary contributions of this paper are:

• Predictive power of network structures: We evaluate topological and centrality-based features for detecting suicidal behavior using traditional machine learning methods, independent of textual content.
• Centrality analysis: We analyze 14 network centrality measures and identify five key metrics that effectively distinguish at-risk users.
• Exploratory multimodal integration: We combine centrality features with SBERT embeddings within a GNN framework as a proof-of-concept investigation into whether structural and textual fusion offers incremental predictive value under constrained data conditions.

## Related work

This section reviews approaches to suicidal ideation detection on social networks, organised into three broad categories: (1) traditional machine learning methods, (2) deep learning techniques, and (3) graph or network-based approaches. We emphasise the latter because, as our study shows, structural network features especially centrality measures remain underexplored. We previously conducted a systematic review of suicidal ideation detection methods published between 2018 and 2024, encompassing 92 peer-reviewed studies across social media, clinical, and multimodal contexts ((Nikmehr, Bilbao-Jayo & Almeida, 2025)). That review identified a significant research gap concerning the under exploration of structural and network-level features, which directly motivates the present work’s focus on centrality-based graph analysis.

Broader reviews of AI methodologies have shown that model performance depends strongly on dataset characteristics, task definitions, and feature representations. Consequently, comparing traditional machine learning methods with graph-based and multimodal approaches remains important rather than assuming that more complex architectures necessarily provide superior performance.

### Traditional machine learning

Early research on suicidal ideation detection primarily used classical machine learning techniques applied to textual content. (Kumar et al., 2020) demonstrated that Random Forest outperformed Logistic Regression and Naïve Bayes in identifying suicidal tweets filtered through keywords such as “die”, “kill myself”, and “end my life”. Similarly, (Goel & Digalwar, 2024) employed a Max Voting Ensemble combining SVM, Logistic Regression, and Random Forest classifiers, improving overall accuracy and robustness by integrating the strengths of multiple models. These methods relied on hand-crafted features such as n-grams and sentiment scores, achieving strong performance on keyword-based corpora. However, such text-centric approaches are limited by their dependence on explicit suicidal expressions and often fail to capture the broader behavioral or relational context of users within a social network.

### Deep learning and transformer-based models

With the advent of deep learning, studies increasingly leveraged neural architectures to capture contextual and semantic nuances. Transformer-based language models such as BERT, RoBERTa, and XLNet have achieved superior results compared to traditional feature-based methods. For instance, (Sawhney et al., 2021) introduced SISMO, a hierarchical attention model that tracks the temporal progression of suicide risk in Reddit posts. Similarly, (Lasri et al., 2024) fine-tuned GPT-3 variants on Reddit data, achieving over 92% F1-score in suicidal ideation classification. (Hasan & Saquer, 2024) compared several transformer architectures and found RoBERTa to perform best in identifying at-risk Reddit users. Recent studies have further explored LLM-based approaches. (Ghanadian, Nejadgholi & Al Osman, 2025) improved performance by augmenting suicidal ideation datasets with synthetic data generated by GPT-3.5, boosting F1-score from 0.87 to 0.91. Although these models demonstrate strong text classification capabilities, they still treat users as isolated entities and do not account for social connectivity or network influence, factors that may carry significant signals of mental distress.

Previous research has shown that machine learning and deep learning approaches exhibit important trade-offs in predictive performance, interpretability, and computational complexity for mental health classification tasks (Ding et al., 2025). Consequently, comparing traditional machine learning methods with graph-based and multimodal approaches remains important rather than assuming that increasingly complex architectures necessarily provide superior performance across all datasets and application settings.

### Graph and network-based methods

A smaller but growing body of research incorporates social network structure into suicidal ideation detection. (Lee et al., 2022) proposed a Contextual Graph Neural Network (GNN) that modeled user posts and words as graph nodes to capture relationships between linguistic and contextual cues. (Naseem et al., 2023) developed a Graph-based Hierarchical Attention Network (GHAN), constructing semantic and syntactic graphs of Reddit posts to integrate local and global features. (Meng et al., 2024) combined social-relationship embeddings with text features to predict latent suicidal risk across platforms, while the Hyperbolic Conversation Network (HCN) ((Sawhney et al., 2022)) modeled hierarchical reply structures in Twitter discussions to capture the dynamics of suicidal conversations. Despite these advances, most graph-based methods focus on text propagation or author profiling rather than analyzing explicit network centrality measures (e.g., degree, betweenness, eigenvector). The quantitative exploration of users’ positions within interaction graphs and how these structural roles relate to suicidal ideation remains limited.

## Data

This study required a dataset containing social media posts that express suicidal ideation, as well as corresponding user interaction data for network analysis. We collected text data from Reddit and then constructed user-level interaction graphs to extract structural features.

### Reddit posts

We collected Reddit posts using the Reddit API, searching across all public subreddits with a list of 25 keywords and phrases related to suicidal ideation. This keyword set was adapted from the lexicon proposed by Sawhney et al., 2018. Table 1 lists the full set of search terms.

**Table 1:** Keywords and phrases indicative of suicidal intent.

suicidal	suicide	my suicide letter
kill myself	slit my wrist	can’t go on
ready to jump	cut my wrist	my suicide note
want to die	sleep forever	slash my wrist
wanna die	wanna suicide	commit suicide
take my own life	thoughts of suicide	I wish I were dead
suicide ideation	suicide plan	end my life
never wake up	tired of living	nothing to live for
go to sleep forever	ready to die	

The keyword lexicon was used strictly as an initial retrieval mechanism rather than a direct labeling criterion. Posts were subsequently manually annotated as suicidal or non-suicidal based on content, allowing the final dataset to include keyword-matched posts that were ultimately labeled non-suicidal. However, because data collection began with explicit suicide-related search terms, this strategy may overrepresent clear linguistic expressions of distress while underrepresenting indirect, metaphorical, or culturally complex expression of suicidal ideation. Consequently, the non-suicidal class contains posts retrieved via suicide-related keywords but judged by annotators to lack suicidal intent; completely non-keyword posts were not sampled. Future work should combine lexicon-based retrieval with semantic search, weak supervision, or broader community sampling strategies to improve representativeness.

Unlike many previous studies that limited data collection to a single subreddit (e.g., r/SuicideWatch), we adopted a platform wide search to reduce topic and community bias, although keyword constraints may still bias toward explicit suicidal discourse.

After removing duplicates and filtering posts containing at least one keyword, we retained 172 Reddit posts published during 2023. For each post, we retrieved up to 15 top-level comments, yielding 298 comments in total. Comments were not keyword-filtered, as they often capture natural conversational responses rather than explicit suicidal expressions (Table 2).

**Table 2:** Distribution of posts and comments.

**Dataset**	**Suicidal**	**Non-Suicidal**
Reddit - Post	82 (48%)	90 (52%)
Reddit - Comments	6 (2%)	292 (98%)

#### Data labeling procedure

We manually labeled all Reddit posts following the definitions of suicidal ideation provided by the World Health Organization (WHO) and the American Association of Suicidology (AAS).

• Posts expressing thoughts, intentions, or plans related to ending one’s life were labeled “suicidal.”
• Posts that mentioned suicide only in general terms or discussed unrelated issues were labeled “non-suicidal.”

While manual annotation provides higher contextual precision than keyword labeling alone, it also introduces potential subjectivity and interpretive bias. Suicidal ideation can be expressed through implicit language, metaphors, sarcasm, or culturally specific phrasing that may not be consistently recognized. Ambiguous cases were excluded when annotators determined that intent could not be reliably inferred from available context. Because disagreements were resolved through discussion, final labels reflect consensus judgments.

Labeling was conducted independently by two annotators with backgrounds in psychology and data science. Inter-annotator reliability prior to reconciliation was assessed using Cohen’s kappa, yielding κ= 0.95, which indicates almost perfect agreement according to standard interpretation guidelines. The annotators disagreed on 4 out of 176 posts, corresponding to an observed agreement of 97.7%. These disagreements involved posts labeled as suicidal by one annotator and non-suicidal by the other. To maintain label quality, Annotation was initially performed on 176 posts. Four ambiguous cases involving disagreement were excluded after reconciliation, yielding a final dataset of 172 posts (82 suicidal, 90 non-suicidal). This high agreement suggests strong consistency in annotation despite the subjective nature of interpreting suicidal ideation in online discourse.

Additionally, the keyword-based retrieval strategy may underrepresent subtle or indirect expressions of distress, particularly those not captured by English language suicide-related terms. Future annotation pipelines should preserve independent pre-reconciliation labels and report formal inter-rater reliability metrics to strengthen methodological transparency.

### Reddit network

**Fig. 1:**
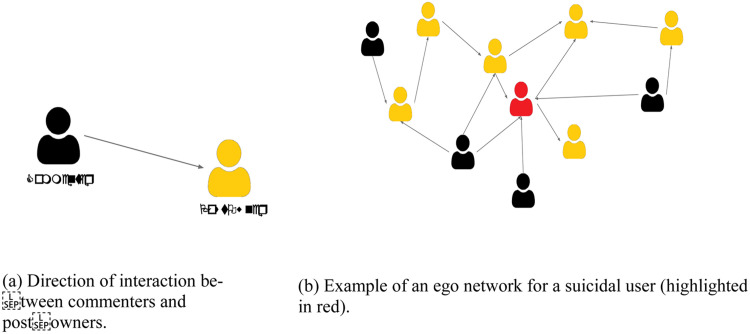
The graph structure is extracted based on each user’s history. In (a), commenters (black) and post owners (orange) are shown, with post owners also having the potential to be commenters. In (b), the ego network of a suicidal user (red) is displayed.

To analyze structural features, we modeled Reddit user interactions as a directed social graph, where nodes represent users and edges represent commenting activity. Specifically, an edge is directed from a commenter to a post owner, indicating a response or interaction (Fig. 1). This structure aligns with the ego-network framework described by Arnaboldi et al., 2016, in which each user (the ego) and their direct connections (alters) form a local subgraph.

To construct a balanced sample, we selected 216 users (118 suicidal, 98 non-suicidal) and extracted their historical posts and comments to build corresponding ego networks. Each user’s graph captures their interaction history and local connectivity patterns.

While the dataset is relatively small to many contemporary machine learning and GNN studies, it provides an exploratory study for investigating whether structural network correlates may contribute to suicide risk detection. Because smaller datasets may increase model variance, overfitting risk, and uncertainty in performance estimates particularly for parameter-rich architectures such as GNNs, our findings should be interpreted as preliminary rather than definitive. Future work should validate these patterns on substantially larger and more diverse datasets. The larger number of users relative to labeled posts reflects the inclusion of interacting users and ego-network participants extracted during graph construction.

After constructing the ego networks, we obtained descriptive statistics summarized in Table 3. In total, the combined set of user graphs includes 2909,864 unique nodes, although some overlap exists because users may appear in multiple ego networks. The graphs vary widely in size, from small networks with only two nodes to large ones exceeding 410,000.

**Table 3:** Summary statistics of generated graphs.

**Metric**	**Value**
Total Graphs	216 (118 Suicidal, 98 Non-Suicidal)
Unique Users	2909,864
Max Nodes	410,600
Min Nodes	2

## Methodology

This section presents our methodological framework, which integrates both textual and structural features to detect suicidal ideation in social networks. Our approach consists of three main components:

• Post-level classification, where we identify suicidal vs. non-suicidal posts (detailed in our prior work (Nikmehr et al., 2025)).
• User network analysis, focusing on graph-structural properties derived from Reddit interactions.
• Graph-based integration, where we combine post-level embeddings with user network features using Graph Neural Networks (GNNs).

**Fig. 2:**
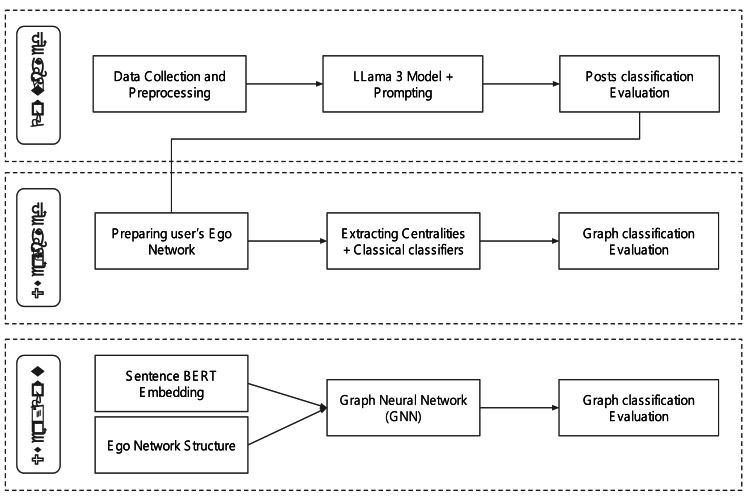
Overview diagram of the methodology.

An overview of the full pipeline is shown in Fig. 2.

### Data preprocessing

Reddit posts often contain noise such as URLs, mentions, and special characters. We apply the following cleaning steps: (1) remove URLs, emojis, and Reddit markdown syntax; (2) normalize user mentions (@username) and remove extra whitespace; (3) exclude non-English posts using langdetect; (4) lowercasing and token normalization.

During data collection, restricted accounts (e.g., ‘[deleted]’, ‘forbidden access’) were excluded. These users and their associated interactions were removed. Comment extraction was limited to 100 per user to reduce API request errors in high-traffic subreddits such as r/AskReddit.

### Post classification

In our companion paper (Nikmehr et al., 2025), we employed Large Language Models (LLMs) with in-context zero-shot prompting for post classification. The model used was LLaMA3-Instruct (Touvron et al., 2023) (8B and 70B), which demonstrated superior performance among tested models. Prompts were optimized to distinguish between Suicidal and Non-Suicidal categories, aligning with WHO and AAS definitions. Each post receives a binary label, and this label is subsequently assigned to the author to form user-level annotations used in later network analysis.

### User network classification

Following post classification, each user is labeled according to their post-level annotations. We then construct ego networks centered on each labeled user, capturing their historical posts and interactions through comments and replies.

### Network definition

Each node represents a Reddit user, and a directed edge from User A → User B indicates that A commented on a post by B. Edges are weighted by the number of interactions between users. We construct ego-1 networks (immediate neighbors only), as higher-order ego expansions (e.g., ego-2) introduced excessive noise and sparse connectivity in preliminary tests. All graphs are directed and unweighted in the final analysis unless otherwise stated.

To mitigate potential bias due to class imbalance (118 suicidal vs. 98 non-suicidal users), we used stratified sampling during data splitting (80/10/10 train/validation/test).

#### Centrality-Based feature extraction

We selected 14 centrality and structural measures based on the taxonomy proposed by Das, Samanta and Pal, 2018, which provides a comprehensive framework encompassing degree, distance, eigenvector, and neighborhood-based centralities. This selection ensures broad coverage of both local and global network properties relevant to social interaction analysis.

• **Degree, In-degree, and Out-degree centrality** – measure user connectivity and engagement.
• **Closeness and Harmonic centrality** – quantify efficiency of information spread.
• **Betweenness centrality** – identifies users bridging communities.
• **Eigenvector, Katz, and PageRank centrality** – capture influence propagation.
• **Clustering coefficient** – measures local cohesion among a user’s connections.
• **Hub/Authority scores (HITS)** – evaluate directional importance within discussion networks.
• **Leverage centrality and Neighborhood centrality** – assess local degree disparity and n-step influence, respectively.

We compute all features using NetworkX 3.3. Feature importance was later evaluated using permutation-based ranking to identify the five most predictive centralities.

#### Traditional classifiers

Traditional classifiers are machine learning models that map input features to predefined categories using mathematical and statistical techniques. These models can be broadly categorized into linear classifiers, probabilistic models, decision trees, and ensemble methods. In this section, after extracting the features, we select classification methods for our analysis. To ensure a comprehensive comparison, we chose a range of models from simple to more complex approaches.

Perceptron (Rosenblatt, 1958) is a basic linear classifier that updates its weights based on misclassified examples, making it suitable for binary classification tasks. Naïve Bayes, introduced by McCallum and Nigam (McCallum & Nigam, 1998), is a probabilistic classifier based on Bayes’ theorem that assumes feature independence and is widely used for text classification.

Support Vector Classifier (SVC) (Cortes & Vapnik, 1995) finds the optimal hyperplane to separate classes, performing well in high-dimensional spaces. CART (Classification and Regression Trees) by Breiman et al. (Loh, 2011) is a decision tree algorithm that recursively splits data based on feature importance. Random Forest (Breiman, 2001) extends this by constructing multiple decision trees to improve accuracy and reduce overfitting.

XGBoost is an optimized gradient boosting framework that iteratively refines decision trees for better predictive performance (Chen & Guestrin, 2016). Finally, MLPClassifier (Multi-Layer Perceptron) is a deep learning model with multiple layers capable of capturing complex patterns in data.

Hyperparameters were tuned via 5-fold cross-validation on the training set. Performance was evaluated using accuracy, precision, recall, and F1-score.

### Integrated graph and text modeling

**Fig. 3:**
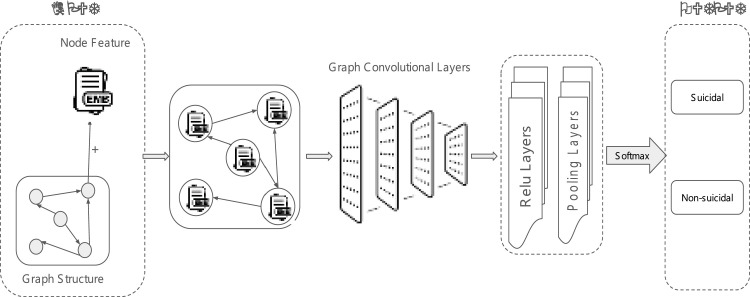
Integration of network structure and text features using GNNs.

The final stage integrates network topology with text-based embeddings for a multimodal approach. We represent each user as a node within the interaction graph and associate it with an embedding vector representing their textual content. The structure of this approach is illustrated in Fig. 3.

#### Graph neural networks

GNNs provide a mechanism to learn joint representations of graph structure and node attributes (Scarselli et al., 2009). In this study, we adopt the GraphSAGE architecture (Hamilton, Ying & Leskovec, 2017) due to its inductive capability and efficiency on large Reddit graphs.

GraphSAGE was selected because inductive architectures can generalize under sparse graph settings and have been applied in low-resource scenarios. The present experiments should be interpreted as feasibility analysis rather than optimization of deep graph learning performance.

#### Model configuration

The configuration of the GraphSAGE-based GNN model used in our experiments is summarized in Table 4.

**Table 4:** Graph neural network (GNN) configuration details.

Parameter	Value / Setting
Architecture	GraphSAGE
Number of layers	2
Hidden dimensions	128 → 64
Aggregator function	Mean pooling
Activation function	ReLU
Dropout rate	0.5
Optimizer	Adam (Kingma & Ba, 2014)
Learning rate	$1\;\times 10^{-3}$
Loss function	Weighted Cross-Entropy
Training epochs	100 (with early stopping after 10)
Graph depth	Ego-1 (directed)
Batch size	32
Framework	PyTorch Geometric 2.5
Hardware	NVIDIA RTX 3090 GPU (24 GB VRAM)

We also experimented with GCN (by Kipf and Welling, 2016) and GAT (by Veličković et al., 2017), but GraphSAGE yielded the best balance of accuracy and computational cost. This configuration follows recommendations from recent studies, such as (Meng et al., 2024) on social risk detection using Graph Attention Networks, and (Hasan & Saquer, 2024) on GNN-based behavioral analysis, which showed the effectiveness of shallow architectures with strong regularization in social contexts.

#### Text embedding: sentence-bert

Each user’s recent posts are concatenated and encoded using Sentence-BERT (SBERT) (Reimers & Gurevych, 2019), producing a 768-dimensional embedding vector. These embeddings serve as node features for the GNN, enriching structural learning with contextual semantics.

SBERT efficiently captures the semantic nuances of user discourse, improving generalization for users with limited posting activity. The embeddings are normalized and projected to 128 dimensions before concatenation with structural features.

#### Evaluation

The GNN outputs a binary prediction per user: Suicidal or Non-Suicidal. Evaluation metrics include accuracy, F1-score, precision, and recall. To ensure reproducibility, all code, configurations, and trained models will be released upon publication.

## Results

### Experimental setup

We designed our experiments with a clear and reproducible setup. For traditional machine learning classifiers, the dataset was split into 70% training, 10% validation, and 20% testing subsets. To ensure reproducibility, we used predefined random seeds (42, 52, 99, and 120). All reported results in Table 5 represent the mean performance across these runs, with standard deviations provided in parentheses.

**Table 5:** Average performance (mean ± SD) across predefined random seeds. Bold values indicate the best score per metric. Non-S. = Non-Suicidal, S. = Suicidal.

Method	Acc.	Prec.	Rec.	F1	ROC AUC	Precision (Class)		Recall (Class)	
						Non-S.	S.	Non-S.	S.
Perceptron	0.52 ₍₀.₀₄₎	0.41 ₍₀.₂₇₎	0.74 ₍₀.₄₉₎	0.52 ₍₀.₃₅₎	0.50 ₍₀.₀₆₎	0.11	0.41	0.25	0.74
Naive Bayes	0.45 ₍₀.₀₂₎	0.33 ₍₀.₄₇₎	0.02 ₍₀.₀₂₎	0.04 ₍₀.₀₄₎	0.49 ₍₀.₀₄₎	0.45	0.33	**0.96**	0.02
SVC	0.50 ₍₀.₀₉₎	0.50 ₍₀.₀₉₎	**0.80** ₍₀.₃₉₎	0.60 ₍₀.₂₂₎	0.46 ₍₀.₀₅₎	0.09	0.50	0.14	**0.80**
CART	0.60 ₍₀.₀₃₎	0.64 ₍₀.₀₄₎	0.61 ₍₀.₁₂₎	0.62 ₍₀.₀₆₎	0.60 ₍₀.₀₅₎	0.56	0.64	0.57	0.61
**Random Forest**	**0.64** ₍₀.₀₇₎	**0.67** ₍₀.₀₅₎	0.66 ₍₀.₁₀₎	0.66 ₍₀.₀₇₎	**0.69** ₍₀.₀₅₎	**0.61**	**0.67**	0.63	0.66
XGBoost	0.60 ₍₀.₀₆₎	0.65 ₍₀.₀₉₎	0.55 ₍₀.₀₄₎	0.60 ₍₀.₀₆₎	0.60 ₍₀.₀₇₎	0.54	0.65	0.64	0.55
MLPClassifier	0.49 ₍₀.₀₂₎	0.53 ₍₀.₀₁₎	0.57 ₍₀.₂₀₎	0.54 ₍₀.₁₀₎	0.54 ₍₀.₀₂₎	0.41	0.53	0.39	0.57
**GNN + SBERT**	**0.67** ₍₀.₀₂₎	0.67 ₍₀.₀₃₎	0.67 ₍₀.₀₃₎	**0.67** ₍₀.₀₂₎	0.67 ₍₀.₀₂₎	0.64	**0.71**	0.69	0.65

Hyperparameter optimization was conducted using Optuna, a Bayesian optimization framework designed for efficient search of parameter configurations. Each model was subjected to 100 optimization trials to identify the best-performing parameters.

For experiments integrating text and network structures via Graph Neural Networks (GNNs), we used the same train–test splits to ensure comparability.

Users without textual data were assigned fixed random embedding vectors generated once and reused across all experiments to preserve dimensional consistency and ensure reproducibility. This approach avoids variability across runs; however, the assigned vectors do not contain meaningful semantic information and may still influence the learned representation. Alternative approaches, such as zero-vector embeddings, masking strategies, or learned placeholder embeddings, should be investigated in future work.

We performed stratified sampling during data splitting to mitigate class imbalance and ensure proportional representation of suicidal and non-suicidal samples across subsets.

### Evaluation metrics

We evaluated models using standard classification metrics: accuracy, weighted precision, recall, F1-score, and ROC AUC. Among these, the F1-score and ROC AUC are emphasized due to their robustness to class imbalance — a common issue in suicide ideation detection.

F1-score provides a harmonic mean between precision and recall, balancing false positives and false negatives.

ROC AUC (Receiver Operating Characteristic – Area Under Curve) assesses the model’s ability to distinguish between positive and negative classes across varying thresholds.

To gain deeper insight, we also report class-specific precision and recall, highlighting how effectively each model detects suicidal users while minimizing false alarms, a critical factor in mental health screening applications.

### Feature selection

Feature selection was performed to enhance interpretability and reduce redundancy among the 14 centrality-based features. All features were first normalized to a [0,1] scale.

**Fig. 4:**
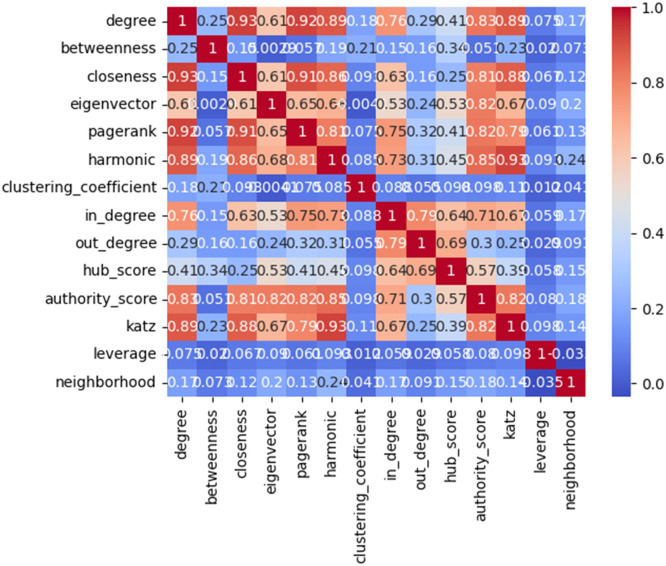
Heatmap plot showing correlations between the 14 features.

A correlation heatmap (Fig. 4) provided an initial overview of relationships among features. Certain measures, such as neighborhood centrality and leverage centrality, showed weak or negative correlations, suggesting they may contribute noise rather than predictive value.

**Fig. 5:**
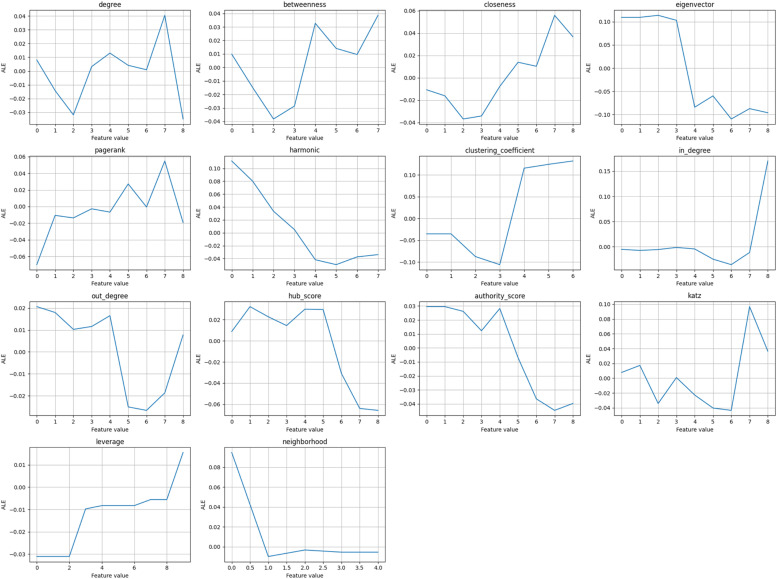
ALE plots showing feature importance in the random forest classifier.

To refine feature importance, we applied two complementary methods:

• Random Forest Feature Importance: Each tree estimates the contribution of a feature to impurity reduction; values are averaged and normalized across the ensemble. This analysis highlighted Degree, Betweenness, Eigenvector, Authority, and Katz centralities as most influential.
• Accumulated Local Effects (ALE): ALE plots quantify the average influence of features on model predictions while controlling for feature correlation bias. As shown in Fig. 5, Degree and Eigenvector centrality exhibit strong positive relationships with suicidal classification likelihood, while Betweenness shows a non-linear effect, peaking at moderate values.

Based on both analyses, we selected the five most informative features, Degree, Betweenness, Eigenvector, Authority, and Katz centrality for the final classification experiments.

### Graph classification results

Using the selected features, we conducted classification using both traditional machine learning models and a GNN-based model that integrates structural and textual features. The averaged results (with standard deviations) are presented in Table 5.

Random Forest achieved the highest ROC AUC (0.69) and F1-score (0.66) among traditional classifiers, demonstrating strong performance and stability. CART followed closely, reflecting the effectiveness of tree-based models in capturing hierarchical feature relationships.

The GNN + SBERT model produced the highest overall accuracy (0.67) and F1-score (0.67), yielding a slight improvement over the strongest traditional baseline (Random Forest, F1 = 0.66). Although paired testing across repeated runs (four random seeds) suggested statistical significance (p < 0.05), these results should be interpreted cautiously because significance estimates derived from a small number of runs and limited datasets may not reliably reflect generalization performance.

Furthermore, given the increased architectural complexity and limited dataset scale, the practical significance of this improvement remains uncertain. Rather than demonstrating substantial predictive superiority, these findings highlight the possibility that integrating structural and textual information may offer incremental value. Given the marginal improvement over Random Forest (ΔF1 ≈ 0.01), the additional computational complexity introduced by multimodal GNN architectures may not be justified under limited-data settings. Simpler models using selected centrality features may provide a more favorable trade-off between interpretability, computational cost, and predictive performance.

Given the sensitivity of inferring suicide risk from online behavior, future deployment of such systems should carefully address fairness and bias, transparency and explainability, privacy and data ethics, and accountability. These considerations are particularly important when behavioral inferences are derived from social network structures, as network-based features may reveal information about users who have not explicitly disclosed mental health concerns. Consequently, any practical use of AI-assisted suicide-risk detection should remain subject to appropriate ethical oversight and human review, consistent with emerging recommendations for trustworthy AI in healthcare (Lekadir et al., 2025).

## Discussion and limitation

Among the network features, Degree centrality proved to be a strong indicator of social connectedness. Users with low degree values exhibit weaker social ties, which may reflect social withdrawal or isolation factors often associated with emotional distress. Betweenness centrality, capturing how often a user serves as a bridge between others, also emerged as significant. Individuals with low betweenness scores tend to occupy peripheral positions within the network, suggesting reduced interaction diversity and potential social marginalization, both of which have been linked to loneliness and increased suicide risk.

Eigenvector centrality and Authority score provide further insight into the quality and influence of a user’s social environment. A low eigenvector score indicates that a user is not connected to well-connected individuals, thereby limiting access to emotionally supportive or resourceful peers. Similarly, a low authority score reflects fewer ties to influential users who could offer advice, empathy, or assistance. Together, these features highlight the importance of both the quantity and quality of social connections in understanding online behavioral health.

Katz centrality, which captures a user’s extended reach through multiple degrees of separation, further reinforces this interpretation. Users with low Katz scores appear socially disconnected even at the network’s broader levels, suggesting limited exposure to supportive communities or interventions. Such users may therefore be more vulnerable to persistent distress, as they remain isolated both directly and indirectly within their social ecosystem.

An additional advantage of centrality-based features is their relative interpretability. Unlike many latent neural representations, centrality measures can be linked to recognizable aspects of online social behavior, such as connectedness, influence, brokerage, and social isolation. This interpretability may support more transparent analysis and facilitate responsible use of AI-assisted mental health research.

Overall, the integration of structural and textual features provides a more comprehensive understanding of user behavior and improves suicide risk detection. Even when analyzed independently, network features capture meaningful behavioral signals, underscoring their value beyond linguistic content.

The predictive performance observed in this study (best F1 = 0.67) is lower than that reported in several recent suicide detection studies using transformer-based architectures on substantially larger datasets. For example, (Lasri et al., 2024) reported F1-scores exceeding 0.90 using fine-tuned GPT-based models, while (Ghanadian, Nejadgholi & Al Osman, 2025) improved performance from 0.87 to 0.91 through synthetic data augmentation.

Many contemporary transformer-based studies are trained on datasets containing thousands of posts or users, providing broader linguistic diversity and greater statistical power for model optimization. In contrast, the present study analyzes 216 users and emphasizes exploratory investigation of structural network features and multimodal integration under constrained data conditions. Therefore, direct performance comparisons remain subject to uncertainty, as differences may reflect dataset scale, sampling strategy, feature modalities, and task formulation rather than solely model capability.

Beyond methodological implications, these insights have real-world relevance. Network-based indicators can support early detection of individuals at risk, even before explicit warning signs appear in text. When coupled with ethical data governance and privacy-preserving mechanisms, such systems could enable collaboration between online platforms and mental health professionals to deliver timely, non-invasive interventions.

However, this study has several important limitations. First, the dataset size (216 users) constrains statistical power and may increase susceptibility to sampling bias, model instability, and overfitting, particularly for complex architectures such as GNNs. Accordingly, this work should be interpreted as exploratory and proof-of-concept rather than conclusive. Second, the initial keyword-based retrieval strategy may bias the dataset toward explicit suicidal expressions, potentially underrepresenting indirect or metaphorical forms of suicidal ideation and limiting generalizability.

Third, although high inter-annotator agreement was observed (κ= 0.95), annotation-based labeling remains susceptible to subjective interpretation, particularly when distinguishing subtle or ambiguous cases. Fourth, the representation of users without textual data remains a methodological limitation that warrants further investigation in future work.

Additionally, because user-level labels were derived from an AI-assisted post-classification process, future work should further evaluate label reliability, potential systematic biases, and agreement with expert human annotation.

In summary, our research highlights that social structure is a powerful yet underexplored dimension of suicide risk detection. When integrated with textual and behavioral cues, graph-based modeling offers a promising pathway toward more holistic, interpretable, and proactive mental health monitoring in online communities.

## Conclusion and future work

In this study, we conducted an exploratory pilot-scale investigation into whether social network centrality measures can complement existing text-based approaches for suicide risk detection on Reddit. By analyzing multiple network centrality measures and evaluating their predictive capacity using traditional classifiers, we assessed how social structure influences suicidal tendencies. Furthermore, we integrated Graph Neural Networks (GNNs) with Sentence-BERT embeddings, enabling a unified model that captures both network connectivity and linguistic context.

Our findings indicate that network structure alone may offer meaningful behavioral insights into suicide risk, even in the absence of explicit textual markers. Features such as degree centrality, betweenness, eigenvector centrality, authority score, and Katz centrality reveal critical aspects of a user’s social connectivity and isolation. However, given methodological constraints including modest sample size, keyword-constrained retrieval, and annotation limitations, these findings should be validated on larger and more diverse datasets before broader claims are made.

Rather than positioning this framework as a state-of-the-art predictive solution, our results suggest that structural network analysis may serve as a complementary and interpretable dimension within broader multimodal suicide detection systems.

Unlike previous studies that focus primarily on textual content, our work underscores the importance of social network analysis in understanding and detecting suicidal ideation. The results indicate that graph-based features enhance detection models, offering a deeper understanding of social behaviors linked to mental health risks. Combining these structural indicators with text features provides a more holistic and explainable framework for identifying individuals at risk.

Looking ahead, future research should explore larger and more diverse datasets, as well as advanced GNN architectures capable of modeling temporal and heterogeneous relationships within online interactions. Integrating psychological and behavioral indicators, such as emotional dynamics, engagement frequency, or community participation, may further strengthen predictive power. Moreover, investigating ethical and privacy-preserving deployment of such models is essential for responsible real-world applications in collaboration with mental health professionals.

These findings underscore the potential of AI-driven social network analysis as a tool for brain health promotion, offering a scalable and non-intrusive means to identify individuals who may benefit from timely psychological support.

Ultimately, this research contributes to a growing understanding that mental health detection should not rely solely on what people say, but also on how they connect. By incorporating both textual and structural perspectives, we move closer to building early-warning systems that are accurate, interpretable, and socially aware, potentially supporting future human-led intervention strategies.

## Ethical approval

This study was reviewed and approved by the institutional ethics committee under the reference number **ETK-05/24–25**.

## Data availability

The data that support the findings of this study are not publicly available due to privacy and confidentiality considerations but may be available from the corresponding author upon reasonable request and with appropriate ethical approvals.

## Declaration of competing interest

The authors declare that they have no competing interests. No financial, professional, or personal relationships were present that might have influenced the research described in this manuscript. All authors approved the final version of the manuscript and agree with its submission to the International Journal of Clinical and Health Psychology.
